# Visualization of Surface Acoustic Waves in Thin Liquid Films

**DOI:** 10.1038/srep21980

**Published:** 2016-02-26

**Authors:** R. W. Rambach, J. Taiber, C. M. L. Scheck, C. Meyer, J. Reboud, J. M. Cooper, T. Franke

**Affiliations:** 1 Soft Matter Group, Lehrstuhl für Experimentalphysik I, Universität Augsburg, Universitätsstr, 1, D-86159 Augsburg, Germany; 2 Division of Biomedical Engineering, School of Engineering, University of Glasgow, Oakfield Avenue, G12 8LT Glasgow, UK

## Abstract

We demonstrate that the propagation path of a surface acoustic wave (SAW), excited with an interdigitated transducer (IDT), can be visualized using a thin liquid film dispensed onto a lithium niobate (LiNbO_3_) substrate. The practical advantages of this visualization method are its rapid and simple implementation, with many potential applications including in characterising acoustic pumping within microfluidic channels. It also enables low-cost characterisation of IDT designs thereby allowing the determination of anisotropy and orientation of the piezoelectric substrate without the requirement for sophisticated and expensive equipment. Here, we show that the optical visibility of the sound path critically depends on the physical properties of the liquid film and identify heptane and methanol as most contrast rich solvents for visualization of SAW. We also provide a detailed theoretical description of this effect.

Surface acoustic waves (SAW), excited and detected by interdigital transducers (IDTs), are used in a wide range of common devices, such as wireless network systems, mobile phones and navigation systems. Other fields of application include gas-sensors^1^, biosensors^2^ and, most recently, total analysis systems and lab-on-a-chip platforms^3^. In microfluidic devices, IDTs are used as actuators^4^,^5^,^6^ for pumping^7^ or mixing of fluids in small volumes^8^,^9^ and manipulation of micrometer sized objects, such as microparticles^10^,^11^,^12^, droplets^13^,^14^,^15^,^16^,^17^ and biological cells^18^,^19^,^20^,^21^ for sorting^22^,^23^,^24^, enrichment^25^,^26^, patterning^27^,^28^ or filtering^29^. The control and manipulation of fluids or drops^30^ on surfaces and the excitation of shear stress in biorheology^31^ are also important applications of SAWs, with great potential in diagnostics, biomedicine and biotech^2^,^32^. However, probing the acoustic propagation in these complex operations is still difficult, often making it impractical. To characterise the acoustic field in these applications, the visualization of SAWs is essential for designing and testing IDTs, probing their functionality and the precise alignment of SAW-chips to other parts of a system (which might for example, include PDMS microfluidic channels^13^,^17^,^28^,^29^). In several applications when IDTs are used as microfluidic actuators, the orientation of the acoustic path or the alignment of the focal point is a critical.

To date, such characterisation tasks remain expensive, time-consuming, and often difficult to perform *in situ*, requiring techniques like laser vibrometry^33^, laser heterodyne interferometry^34^,^35^, scanning acoustic force microscopy^36^, phononic crystals^37^, stroboscopic topography setups^38^ or direct photoluminescence at very low temperatures^39^. These techniques do not allow direct access to closed systems, as would be the case inside a channel, and are impractical for a rapid analysis and probing of IDTs for an experimental setup. Also smoke particles have been used for visualization^40^ of acoustic streaming in air. Here, we present a rapid method for the direct and simple visualisation of SAWs using a wetting fluid film deposited onto the piezoelectric substrate (Fig. 1). The interaction of the acoustic wave with the fluid film gives rise to film deformations and undulations induced by the acoustic radiation pressure and causes a visible optical contrast between excited and non-excited sections of the film. Observed with a conventional light microscope the optical contrast of the sound path is dependent on the speed of sound, as well as the density and surface tension of the used fluid. The method not only enables the visualization of the sound path itself for different IDT designs, such as Tapered IDTS (TIDTs), focusing IDTs and tapered focusing IDTs, but it also provides access to the speed of sound of a SAW and the crystals anisotropy. This method could accelerate the development of SAW-based lab-on-a-chip systems and their broad application in life science.

## Results

**Figure 1:**
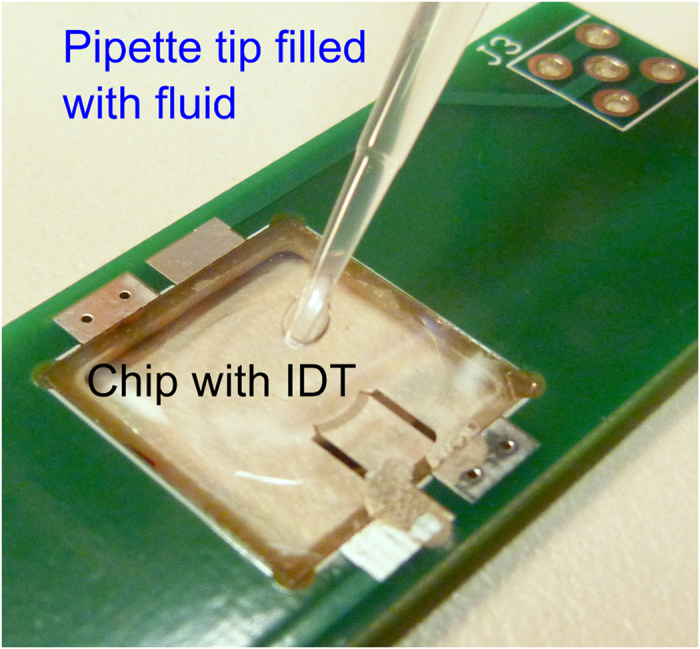
Experimental set-up, where the transparent LiNbO_3_ chip is shown with a focusing IDT mounted on a circuit board (green). The chip is wetted with a liquid (in this case isopropanol) creating a thin fluid film covering the chip.

**Figure 2:**
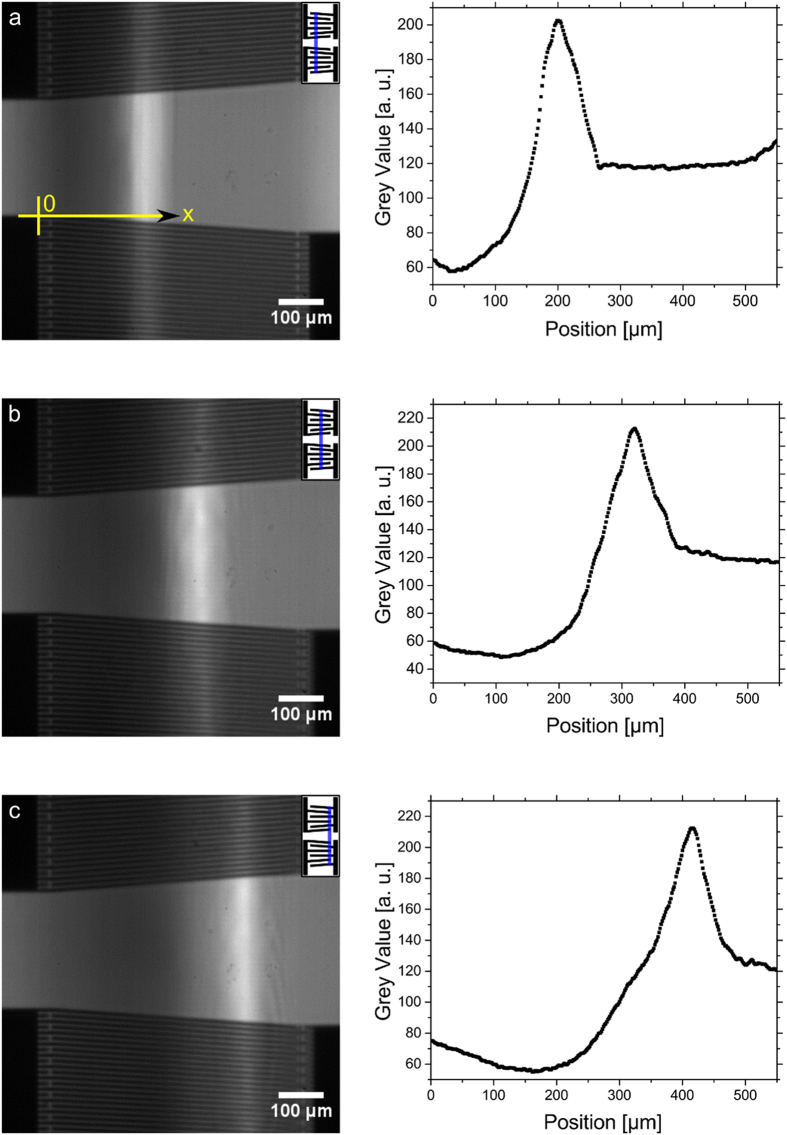
Two opposed tapered IDTs at 163 MHz (**a**), 165 MHz (**b**) and 167 MHz (**c**). The acoustic path of the SAW is shifted with frequency (white region in the left micrograph panel). The grey value is measured and plotted along the x-axis in the corresponding graphs on the right. The diagrams reveal the positions of the acoustic path for the three applied frequencies. Small insets in the micrographs show the orientation of the two TIDTs and the relative position of the acoustic path (blue line).

In order to analyse an IDT, we deposited a microliter volume of liquid onto the chip using a pipette tip to wet the chip completely with a thin film as shown in Fig. 1. By applying a high-frequency signal, the IDT excites surface acoustic waves (Rayleigh waves). The SAW couples into the liquid and converts into a longitudinal wave and the propagation path of the SAW becomes visible (formation of a characteristic pattern in ~10 ms) (Fig. 2).

### Position of acoustic path

First we excited two opposed tapered TIDTs (with wavelengths from 23 to 24.3 μm, 60 finger-pairs and 500 μm aperture) at different frequencies and isopropanol for the liquid layer (Fig. 2). The resonance frequency f of an IDT is given by f = c/λ, with the propagating velocity c of the SAW and the wavelength λ (finger distance equals λ/2). Switching the frequency changed the position of the SAW, as the finger distance varies along the aperture.

**Figure 3:**
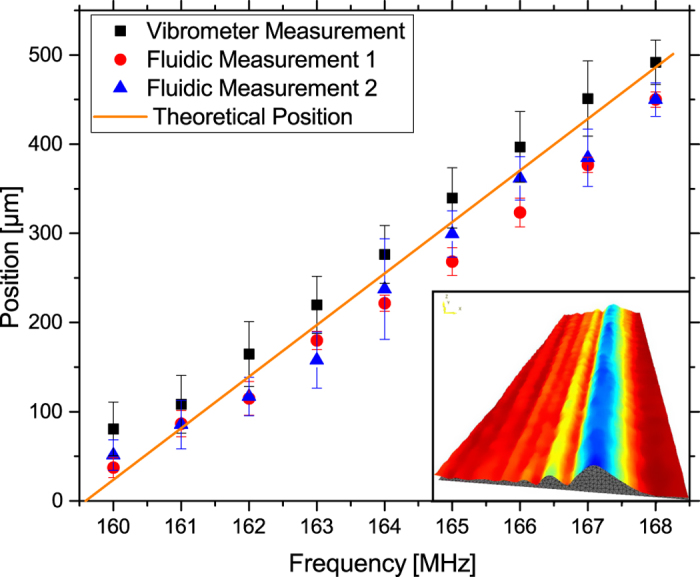
Comparison of the position of the acoustic path determined from our new fluidic visualization method, vibrometer measurements and calculated values (with SAW velocity of 3870 m/s^41^). The position of the acoustic path is shifted linearly with the frequency. The slopes of the fit for each method are in good agreement with each other: 51.06 ± 1.45 μm/MHz (fluidic measurement 1), 51.36 ± 1.94 μm/MHz (fluidic measurement 2), 53.62 ± 1.18 μm/MHz (vibrometer measurement) and 57.79 μm/MHz (theory). The SAW velocity is slightly changed by the thin liquid film, which is reflected by the small offset between fluidic measurements and theory. The intensities of the acoustic path were fitted with a Gaussian function and the errors are given by half width of the half maximum (1/2 FWHM). The inset shows an exemplarily vibrometer measurement.

To verify our new method, the position (along the x-axis, see Fig. 2a) of the acoustic paths of two excited TIDTs of the same geometry were measured at different frequencies, fitted by a Gaussian function, and compared with vibrometer measurements and theory (Fig. 3). The theoretical values were calculated by the given finger distances of the TIDT and the theoretical SAW velocity of 3870 m/s^41^. A convincing agreement between the two measurement methods and theory was obtained. The linear shift of the sound paths´ position with increasing frequency is clearly visible. The slopes of each liner fit were measured to 51.06 ± 1.45 μm/MHz (fluidic measurement 1), 51.36 ± 1.94 μm/MHz (fluidic measurement 2), 53.62 ± 1.18 μm/MHz (vibrometer measurement) and 57.79 μm/MHz (theory). The systematic off-set between the theory and experiments can be explained by the change of the SAW velocity: The electrodes of the IDT decreased the velocity from 3978 m/s to 3870 m/s as piezoelectric shorting has to be included^41^. But this effect was reduced by the presence of an additional dielectric medium on the substrate, resulting in a higher SAW velocity than the theoretical one in the case of isopropanol covered SAW-chip^42^,^43^,^44^,^45^,^46^.

**Figure 4:**
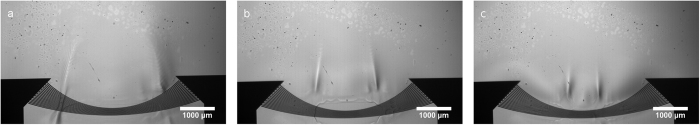
Micrographs of a tapered focus IDT excited at three different frequencies ((**a**) 120 MHz, (**b**) 180 MHz, (**c**) 240 MHz). With higher frequencies the acoustic path shifted towards the centre of the IDT. The surface velocities, which depend on direction of travelling, as LiNbO_3_ is an anisotropic substrate, for each of the three positions (**a–c**) can be determined to 3800 ± 400 m/s, 4000 ± 710 m/s and 3700 ± 600 m/s. The experimental values are in good agreement with the literature values of 3772 m/s, 3830 m/s and 3864 m/s^41^.

We used this new visualization method to determine the velocity of the SAW on the piezoelectric substrate accordingly. The wavelength λ of a TIDT at the excited frequency f can be measured, using the visible sound path position. The results shown in Fig. 2 lead to velocities of 3823 ± 48 m/s, 3807 ± 49 m/s and 3822 ± 66 m/s. The measured values are within 2% of the literature value for LiNbO_3_ of 3870 m/s (including only the piezoelectric shorting and neglecting influence of the isopropanol layer)^41^. To demonstrate the applicability of the technique in characterising more complex IDT systems, we next used a tapered focusing IDT: in addition to a conventional focusing design, the fingers are slanted as in the case of a tapered IDT. The IDT was excited at different frequencies. As before, the position of the acoustic path changed with frequency (Fig. 4). The average wavelength for each position was measured. With these values the SAW velocity was calculated to be 3800 ± 400 m/s, 4000 ± 710 m/s and 3700 ± 600 m/s. As LiNbO_3_ is an anisotropic substrate, the velocity of sound varies with the direction of travel. The experimental values are in good agreement with the literature values of 3772 m/s, 3830 m/s and 3864 m/s for the different angles at each of the positions (piezoelectric shorting is included in these velocities)^41^.

### Measurement of anisotropy and focal point

**Figure 5:**
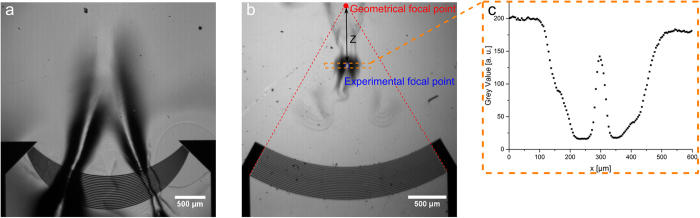
(**a**) The two symmetric acoustic paths excited by a single tapered focusing IDT at 90 MHz are visualized. Both paths hit in the focal point. (**b**) The difference of the geometrical focal point (as given by the centre of the concentric, circularly aligned electrodes) and the experimentally found focussing point (as given by the focus of the fluid) is demonstrated. Due to the anisotropy of LiNbO_3_ and different SAW velocities in different directions, the experimental focal points is shifted towards the IDT^48^ with respect to the geometrical focal point. (**c**) Measured grey value in the orange-marked region of b (averaged in vertical direction).

As well as TIDTs, focusing IDTs are used as actuators in microfluidics for producing and merging droplets, transport and alignment of particles and other applications^13^,^17^,^47^. In one application it is necessary for alignment of a PDMS microchannel with respect to the orientation of the IDT, especially where considering the focus point of the IDT and the anisotropy of the piezoelectric substrate. To demonstrate this practical necessity and determine appropriate parameters, the sound path of a focusing IDT (wavelength of 60 μm, opening angle of 60°, 20 finger-pairs, and radius R of 3 mm) and a tapered focusing IDT were characterised (Fig. 5). Again, isopropanol was used for wetting the chip. The acoustic path and the focal point were clearly visible. The piezoelectric substrate LiNbO_3_ and hence the velocity of the SAW was anisotropic, leading to a shift Z = 900 ± 77 μm of the focal point towards the IDT for the focusing IDT (Fig. 5b). The value determined optically again was in good agreement with the theoretical value Z = 940 μm^48^. From the measured distance Z between geometrical and real focal point, the anisotropy factor b can be calculated with the formula by Green *et al.*^48^:


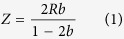


The literature value^41^ for the SAW propagation velocity was fitted with a parabolic function 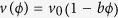
48 with the velocity 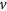, the propagation angle 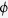 towards the x direction and the anisotropy factor b. The parabolic fit leads to a theoretical value b_fit_ = −0.228 (with dimensions of degree^−2^). Our simple measurements deliver an anisotropy factor b_exp_ = −0.216 ± 0.026. The deviation between theoretical and experimental values was less than 6%.

### Influence of different fluidic layers

**Figure 6:**
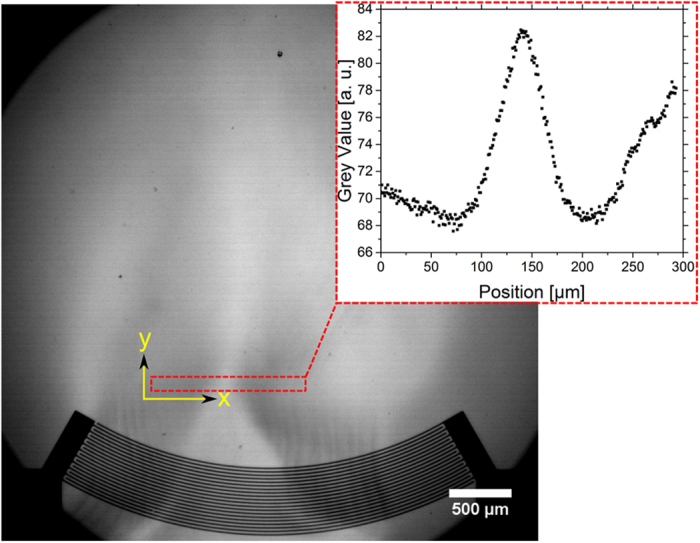
Micrograph of a thin heptane film layer. To analyse the sound path the grey value in the red-marked region in front of the IDT is averaged in vertical direction (y-axis). A plot of the grey value depending on the x-axis (horizontal position) is shown in the graph.

**Figure 7:**
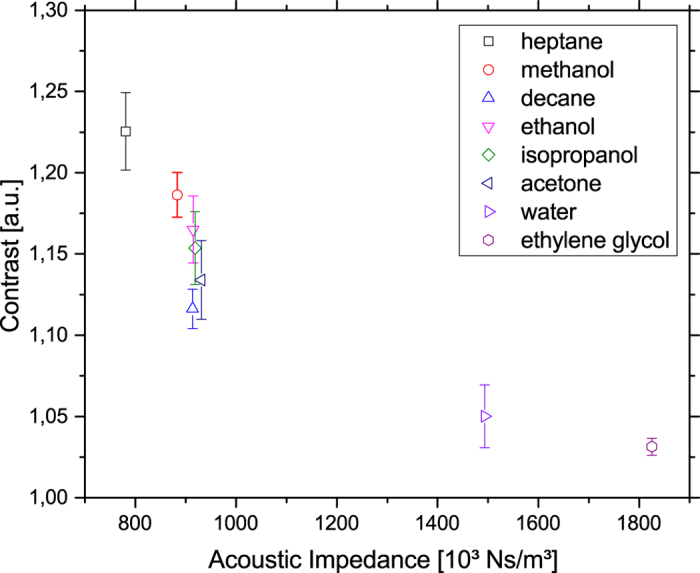
Optical contrast as given by the ratio of the grey values of the maximum and the baseline plotted against the acoustic impedance for different fluids. The optical contrast decreases with higher acoustic impedance. Experimental points show the mean value with standard deviation.

To explore the dependence of the effect on the acoustic properties of the fluid layer, different solvents were used in a series of experiments (including heptane, methanol, decane, ethanol, isopropanol, acetone, water and ethylene glycol). The experimental set-up involved using a single focusing IDT, as described above. All experiments were performed with the same power of 18 dBm. A contrast increase with experimental time is caused by evaporation of the fluid. To avoid the influence of the thickness of the fluid layer over time (where a contrast increase occurs with decreasing thickness of the fluid layer) a standardized volume of liquid (20 μl) was dispensed on the whole area of the chip. A single image was extracted of the video shortly (<1 s) after application of the RF-signal. To determine the optical contrast, the grey value in the area in front of the IDT was averaged in vertical direction (Fig. 6) and the ratio of maximum and bottom line of the peak is measured. This ratio is independent of the type of fluids used and is therefore a practical experimental parameter for examining the relation between the acoustic actuation and the optical observation between different fluids. Figure 7 shows this optical contrast as function of the acoustic impedance. As expected, the optical contrast is raising with decreasing acoustic impedance of the fluid.

Therefore heptane and methanol were the most suitable fluids for visualizing the SAW with this new method, in that they provided the highest contrasts.

## Discussion

In order to explain the optical contrast in the experiments, the coupling and reflection of the SAW has to be understood. The excited SAW on the substrate couples into the fluid under the Rayleigh angle 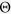 5,49:


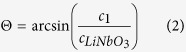



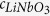
 and 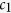 are the acoustic wave velocities of the piezoelectric substrate LiNbO_3_ and the fluid, respectively. Since the acoustic wave velocity on LiNbO_3_ was more than two times larger than the velocity in the fluid, the Rayleigh angle is 30° or lower, most of the acoustic pressure is pointing towards the surface of the fluid. A large acoustic impedance mismatch exists at the interface of the fluid and air, resulting in reflection of most of the acoustic energy. The reflection coefficient R was given by^49^:


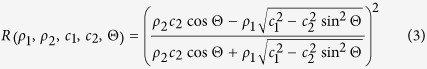



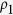
 and 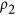 being the densities and 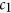 and 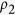 the speeds of sound of the fluid and air. The incoming, reflected and transmitted acoustic waves cause an acoustic radiation pressure p onto the surface, given by^49^:


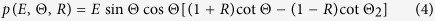


There by 
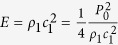
 is the energy flux density in the fluid, with 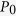 being the amplitude of the acoustic pressure field of the sound wave^50^,^51^. The angles 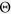 and 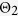 of the incoming and transmitted waves are linked by the relation^49^:


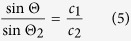


The acoustic radiation pressure at the surface leads to a deformation of the fluidic surface and causes an optical contrast between excited and non-excited sections of the film visible with the microscope^52^,^53^. We assume, the deformations induced by the acoustic pressure are related to the curvature of the surface by Laplace’s law and causes the different optical contrasts. We observed that the SAW leads to a deformation, however we cannot give an exact quantitative relation between deformation and contrast.

As an example for a qualitative explanation, the acoustic path of a focus IDT is visualized (Fig. 5b) and the grey value for the orange marked is plotted (Fig. 5c). The acoustic radiation pressure at the focal point is maximal and the curvature of the fluid surface is convex and resulting in a bright spot. The fluid close to this point is curved concave, leading to a darker area. The observed contrast depends on the acoustic pressure and the resulting curvature.

**Figure 8:**
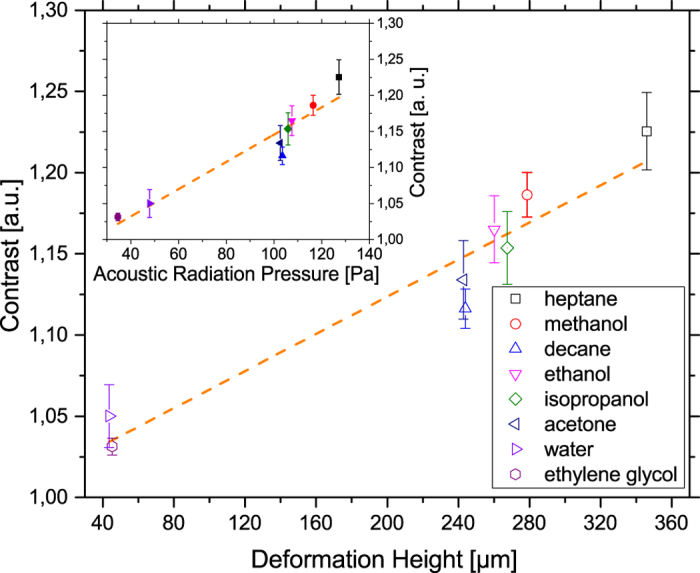
Optical contrast against calculated deformation height with a roughly estimated acoustic pressure amplitude of 0.5 MPa. The optical contrast raises with larger deformation height. Inset: Optical contrast against calculated acoustic radiation pressure. The optical contrast decreases with lower acoustic radiation pressure as the deformation of the fluid is also reduced with lower pressure. Experimental data shows the mean value with standard deviation. The orange dashed lines are guides to the eye.

Inset of Fig. 8 shows the optical contrast for different fluids as function of the calculated acoustic radiation pressure. The amplitude of the pressure field 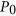 is not known but constant for all experiments, as the applied power is kept the same. It is roughly estimated by 0.5 MPa (
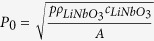
 with the applied power p and the area A^54^,^55^). Values for the physical properties of the different fluids are taken from^56^,^57^. The greatest acoustic radiation pressure is achieved with heptane and methanol. In the experiments these fluids also give the greatest optical contrast and are therefore the most suitable fluids for visualizing the SAW with this new method.

This explanation is based on the observations and explanations by Issenmann *et al.*, who examined deformation caused by acoustic radiation pressure in their works^52^,^53^. They used a focusing, spherical ultrasonic transducer to excite bulk acoustic waves in the fluid to deform a liquid-liquid and liquid-air interface in the focal plane of the transducer. The surface deformation is stationary for moderate acoustic intensities. The authors also described their observations with the theory of Langevin and a one-dimensional model of a compliant Fabry-Pérot resonator. By studying the possibility of mechanical and acoustic streaming effects on the deformation, they concluded that these effects are negligible in comparison to the acoustic radiation pressure. Thus these effects could also be excluded from the theoretical explanation for our experiments.

They found that the deformation height is a stationary state, where acoustic radiation pressure, gravity forces and surface tension are in balance. The deformation height depends on the pressure amplitude and applied frequency of the transducer and on the properties of the fluid: surface tension 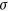, density 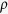 and speed of sound 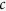. The deformation height h in the centre of the acoustic path for a perpendicular incidence of the SAW is given by:


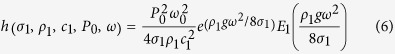


*E*_1_ is the first argument of the exponential integral function. *ω* is the beam waist, roughly approximated by three wavelengths in our experiments (3∙60 μm) and g the gravitational constant. The acoustic pressure amplitude 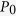 is roughly estimated by 0.5 MPa, as before. The deformation height seems responsible for the optical contrast in our experiments. With higher radiation pressure, the deformation height increases as well as the observed optical contrast (Fig. 8).

For our experiments the Bond number 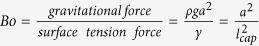 with the characteristic length a, as approximated by the maximum deformation height of 400 μm given by the equation (6) and Fig. 8, and the capillary length 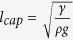, could be estimated to be in the regime of 8∙10^−3^ to 21∙10^−3^ 58. Therefore, the surface tension always dominates over the gravitation for all examined fluids in our experiments.

### Experimental setup and methods

We used different designs to excite the SAW (tapered IDTs, focusing IDTs and a combination of both). To fabricate the IDTs, layers of 10 nm Ti, 50 nm Au and 10 nm Ti were deposited onto a 17.5 mm x 17.5 mm piezoelectric substrate (128° y-cut LiNbO_3_) via electron beam evaporation. The specific cut is chosen to ensure that only Rayleigh waves (transversal surface acoustic waves with a small longitudinal part) are excited by the IDT and that other modes are almost completely supressed. Optionally, a 200 nm thick SiO_2_ layer was sputtered onto the LiNbO_3_ chip with the IDT for mechanical protection. The device was connected to a frequency generator (SML-01, SMB100A or SMP02 by Rhode & Schwarz) and mounted on a microscope (Axiovert 200M by Carl Zeiss, Germany (numerical aperture of 0.25; 2.5, 4x or 10x objective). For the illustrating Fig. 2 a CKX41 microscope by Olympus, Germany, with a fixed condenser and a 10 x objective was used.). We used bright-field illumination microscopy in all experiments. High power output was used to enhance visualization (18 dBm to 25 dBm), using an amplifier ZHL-2 (Mini-Circuits). The images were recorded with a high-speed camera (Fastcam 1024 PCI, Photron) using a frame rate between 125 fps and 1000 fps. To improve the wettability, we treated the surface of the chips with an O_2_-plasma. For evaluating the optical contrast the software ImageJ^59^ and Fiji^60^ were used. For better visibility brightness and contrast were changed for some presented images.

## Additional Information

**How to cite this article**: Rambach, R. W. *et al.* Visualization of Surface Acoustic Waves in Thin Liquid Films. *Sci. Rep.* **6**, 21980; doi: 10.1038/srep21980 (2016).
