# Corrigendum: Visualization of Surface Acoustic Waves in Thin Liquid Films

**DOI:** 10.1038/srep24962

**Published:** 2016-05-13

**Authors:** R. W. Rambach, J. Taiber, C. M. L. Scheck, C. Meyer, J. Reboud, J. M. Cooper, T. Franke

Scientific Reports 6: Article number: 2198010.1038/srep21980; Published online: 02262016; Updated: 05132016

This Article contains errors in the Results section under subheading ‘Measurement of anisotropy and focal point’,

“The literature value^41^ for the SAW propagation velocity was fitted with a parabolic function 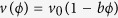
^48^ with the velocity *v*, the propagation angle *ϕ* towards the x direction and the anisotropy factor b. The parabolic fit leads to a theoretical value b_fit_ = −0.228 (with dimensions of degree^−2^)”.

should read:

“The literature value^41^ for the SAW propagation velocity was fitted with a parabolic function 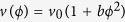
^48^ with the velocity *v*, the propagation angle *ϕ* (in radian) towards the x direction and the anisotropy factor b. The parabolic fit leads to a theoretical value b_fit_ = −0.228 (b is dimensionless)”.

